# Release of tumour necrosis factor alpha into bronchial alveolar lavage fluid following antigen challenge in passively sensitized guinea-pigs

**DOI:** 10.1155/S0962935192000644

**Published:** 1992

**Authors:** D. E. Kelly, M. Denis, D. F. Biggs

**Affiliations:** 1 Faculty of Pharmacy and Pharmaceutical Sciences University of Alberta Edmonton Alberta T6G 2N8 Canada; 2 pulmonary Research Unit Faculty of Medicine University of Sherbrooke Sherbrooke Québec J1H 5N4 Canada

## Abstract

Five groups of ten female guinea-pigs were passively sensitized against ovalbumin (OA) (n = 9) or control guinea-pig serum (n = 1). 24 h later, they received mepyramine (0.5 mg/kg, i.p.) and 30 min later inhaled aerosols of: (A) OA (2 in 0.9% saline, 8 min, n = 4/9); (B) saline (40 min, n = 4/9); (C) LPS (40 min, Escherichia coli 0111:B4, 150 ng/kg in PBS, n = 1/9); and (D) the control animal was treated as in (C) (n = 1). Their tracheas were cannulated under pentobarbital anaesthesia and bronchial alveolar lavage (BAL) was performed with 2 × 5 ml PBS containing BSA (1%) (n = 1 group), or BSA (1%) and aprotinin (1000 KIU/ml) (n = 4 groups), at 30, 60, 90 or 120 min post-inhalations. BAL fluids recovered were centrifuged, the supernatants recovered and frozen until assayed for tumour necrosis factor-α (TNF-α), interleukin-1 (IL-1) and interleukin-6 (IL-6). No TNF-α could be detected unless aprotinin was present in the lavaging solution. BAL fluid from OA-sensitized and control animals that had inhaled LPS contained high levels of TNF-α that peaked at 90 min. BAL fluid from OA sensitized animals that inhaled OA aerosols contained no detectable TNF-α at 30 min, but it was found in increasing amounts at 60, 90 and 120 min; TNF-α was not detected in fluid from any of the animals that inhaled saline. As BAL fluids were toxic to the cells used in the assays, neither IL-1 nor IL-6 could be measured. We conclude that the monokine TNF-α is released into BAL fluid following anaphylactic challenge of passively sensitized guinea-pigs. The presence of the antiprotease, aprotinin, in the lavaging solution is essential for the detection and measurement of TNF-α in BAL fluid.


Mediators of Inflammation 1, 425–428 (1992)

### Release of tumour necrosis factor alpha into bronchial alveolar lavage fluid following antigen challenge in passively sensitized guinea-pigs

D. E. Kelly,[1] M. Denis[2] and D. F. Biggs[1,CA]

[1] Faculty of Pharmacy and Pharmaceutical Sciences, University of Alberta, Edmonton, Alberta, Canada T6G 2N8 and [2] Pulmonary Research Unit, Faculty of Medicine, University of Sherbrooke, Sherbrooke, Québec J1H 5N4, Canada

[CA] Corresponding Author

**FIVE groups of ten female guinea-pigs were passively sensitized against ovalbumin (OA) ($n = 9$) or control guinea-pig serum ($n = 1$). 24 h later, they received mepyramine (0.5 mg/kg, i.p.) and 30 min later inhaled aerosols of: (A) OA (2% in 0.9% saline, 8 min, $n = 4/9$); (B) saline (40 min, $n = 4/9$); (C) LPS (40 min, *Escherichia coli* 0111:B4, 150 ng/kg in PBS, $n = 1/9$); and (D) the control animal was treated as in (C) ($n = 1$). Their tracheas were cannulated under pentobarbital anaesthesia and bronchial alveolar lavage (BAL) was performed with 2 × 5 ml PBS containing BSA (1%) ($n = 1$ group), or BSA (1%) and aprotinin (1000 KIU/ml) ($n = 4$ groups), at 30, 60, 90 or 120 min post-inhalations. BAL fluids recovered were centrifuged, the supernatants recovered and frozen until assayed for tumour necrosis factor-α (TNF-α), interleukin-1 (IL-1) and interleukin-6 (IL-6). No TNF-α could be detected unless aprotinin was present in the lavaging solution. BAL fluid from OA-sensitized and control animals that had inhaled LPS contained high levels of TNF-α that peaked at 90 min. BAL fluid from OA sensitized animals that inhaled OA aerosols contained no detectable TNF-α at 30 min, but it was found in increasing amounts at 60, 90 and 120 min; TNF-α was not detected in fluid from any of the animals that inhaled saline. As BAL fluids were toxic to the cells used in the assays, neither IL-1 nor IL-6 could be measured. We conclude that the monokine TNF-α is released into BAL fluid following anaphylactic challenge of passively sensitized guinea-pigs. The presence of the antiprotease, aprotinin, in the lavaging solution is essential for the detection and measurement of TNF-α in BAL fluid.**

**Key words:** Anaphylaxis, Bronchial alveolar lavage, Guinea-pig, Interleukin-1, Interleukin-6, Passive sensitization, Tumour necrosis factor-α

### Introduction

Cytokine mediated interactions among macrophages, lymphocytes and eosinophils appear to be involved in the pathogenesis of the eosinophilia and airways' inflammation that characterize asthma.[1–4] Thus, tumour necrosis factor alpha (TNF-α) and granulocyte-macrophage colony-stimulating factor (GM-CSF), given parenterally, induce the accumulation of eosinophils in the airways of normal guinea-pigs.[5] Also, TNF-α is released from sensitized lung tissue following IgE receptor triggering,[6] and it contributes to mast cell dependent recruitment of leukocytes during IgE dependent cutaneous late phase reactions.[7] Alveolar macrophages isolated from bronchial alveolar lavage (BAL) fluid from allergen challenged patients undergoing a late asthmatic response secrete greater amounts of TNF-α and interleukin-6 (IL-6) than macrophages isolated from BAL fluid of patients who develop no response or only an early response to allergen challenge.[8] However, Gosset *et al.*[8] were unable to detect TNF-α in BAL fluid from asthmatic patients challenged with antigen inhalation. By contrast, Broide *et al.*[9] showed that levels of several cytokines, including TNF-α, IL-1 and IL-6, were significantly elevated in BAL fluid from patients with symptomatic asthma compared to asymptomatic asthmatic controls. We used guinea-pigs passively sensitized to ovalbumin (OA), and determined whether the cytokines TNF-α, interleukin-1 (IL-1) and interleukin-6 (IL-6) could be detected in BAL fluid immediately after challenge with an aerosol of OA. Guinea-pigs passively sensitized to OA and to inert serum that inhaled LPS served as positive controls for TNF-α release.

### Methods

*Animals:* Five groups of ten female Hartley strain guinea-pigs, SPF quality, weight range 320–400 g, obtained from Charles River, St Constant,

© 1992 Rapid Communications of Oxford Ltd

Québec were housed in laminar flow units (Bioclean™, Hazleton, MD) on grids in cages suspended over trays of rock salt. They were fed normal guinea-pig chow supplemented with apples and allowed water *ad libitum*. They were monitored for at least one week after being shipped to ensure that they were in good health. They weighed 350–450 g at experiment.

*Experiments:* In each of the five groups of ten guinea-pigs, nine were passively sensitized to OA with guinea-pig hyperimmune serum (anti-OA antibody titre = 1:5200, by ELISA; 0.2 ml, i.p.), and one animal received control guinea-pig serum (0.2 ml, i.p.). One day later, all animals received mepyramine (0.5 mg/kg, i.p.) and 30 min later, the nine guinea-pigs that had received hyperimmune serum inhaled aerosols (Vix Acorn™ nebulizer, compressed air at 10 psi) of: (A) OA (2% in 0.9% saline, up to 8 min, $n = 4/9$); (B) 0.9% saline (as for (A) $n = 4/9$); (C) LPS (*E. coli* 0111:B4, 150 ng/ml in PBS, 40 min, $n = 1/9$; and (D) the guinea-pig that had received control serum was treated as in (C). At 30, 60, 90 and 120 min after challenge with OA or LPS, the groups of ten animals were anaesthetized (pentobarbital, 40–50 mg/kg, i.p.), their tracheas cannulated (PE240) and BAL performed.

*Bronchial alveolar lavage:* One group of guinea-pigs' lungs was lavaged with 2 × 5 ml PBS containing BSA (1%); the other four groups were lavaged with 2 × 5 ml PBS containing BSA (1%, to maintain viability of any cells recovered) and aprotinin (1000 KIU/ml, to inhibit protease activity[8]). BAL fluids recovered were centrifuged (2000 rpm, 5 min) and the supernatant removed and immediately frozen at −20°C until assay.

*Cytokine measurements:* All assays were performed in triplicate. Levels of TNF-α were measured by the specific ability of this cytokine to exert cytotoxicity against the L929 fibroblast cell line.[10] Briefly, L929 cells ($10^5$) in 100 μl of complete medium (RPMI 1640, 10% FBS, 1% penicillin-streptomycin) and actinomycin D (1.0 μl/well) were added to serial dilutions of BAL fluids and incubated for 18 h (37°C, 5% $CO_2$). Then, 3-(4,5-dimethylthiazol-2-yl)-2,5-diphenyltetrazolinium bromide (MTT, 25 μl) was added to each well. After further incubation (4 h), acidified isopropanol was added and optical densities read at 570 nm. Concentrations of TNF-α in the lavage fluids were calculated using probit analysis from a standard curve prepared using human recombinant TNF-α.

IL-6 was measured as described recently.[11] Briefly, B9 hybridoma ($10^5$ cells/well) was incubated in fortified RPMI 1640 medium, in the presence of serial dilutions of BAL fluid. Growth stimulation was assessed by the MTT procedure described above. IL-6 levels were derived from a standard curve prepared with human rIL-6.

IL-1 was measured via the ability of samples of BAL fluid to support the concanavalin A driven growth of D10.G4.1 cells.[12] Cells ($4 \times 10^5$) were suspended in complete medium containing concanavalin A (2.5 μg/ml) with serial dilutions of BAL fluid. After incubation (72 h, 37°C, 5% $CO_2$), growth was assessed by the MTT procedure. A standard curve was prepared using human recombinant IL-1.

*Guinea-pig sera:* On day 0, guinea-pigs were given OA (20 mg/kg, i.p.); 21 days later, they inhaled OA aerosols (2% in saline, mepyramine, 0.5 mg/kg, i.p., 30 min beforehand) for up to 8 min, on 8 consecutive days. On day 35, they were anaesthetized (pentobarbital, 50 mg/kg, i.p.) and blood collected into plain glass tubes via cardiac puncture. Tubes were centrifuged and stored at 4°C overnight to allow the clot to form and retract. Serum was collected, pooled and stored at −20°C until use. Anti-OA antibody titres, as total IgG, were estimated via ELISA. Control sera were prepared similarly except animals received only vehicle on day 0, and inhaled only saline aerosols. No anti-OA antibodies could be detected in control sera.

*Reagents and chemicals:* Actinomycin D, aprotinin, concanavalin A, 3-(4,5-dimethylthiazol-2-yl)-diphenyltetrazolinium bromide (MTT), chicken egg albumin (ovalbumin, Grade V) and LPS (*E. coli* 0111:B4) (Sigma, St Louis, MO), penicillin-streptomycin mixture and RPMI 1640 medium (Gibco, Grand Island, NY) FBS (PA Biologicals, Sydney, Australia), recombinant human TNF-α and human recombinant IL-1, (Amersham Canada Ltd, Oakville, Ontario), rabbit anti-human TNF-α and pre-immune serum (Olympus Corp., New York, NY), and human recombinant IL-6 (British Biotechnology, Oxford, UK).

*Statistical analyses:* Data were expressed as mean ± S.E.M. Differences among the groups were examined using Student's *t*-test. Differences were assumed significant at the 5% level.

### Results

*TNF-α:* In the absence of aprotinin, TNF-α could not be detected in BAL fluids obtained at 60 min post-challenge whether guinea-pigs had inhaled OA saline or LPS (positive controls) aerosols. However, if animals' lungs were lavaged with fluid containing the antiprotease, aprotinin (1000 KIU/ml), significant amounts of TNF-α were detected (Table 1, Fig. 1). In the group challenged with OA aerosol, TNF-α was not detectable in BAL

**Table 1**. Amounts of TNF-α detected in bronchial alveolar lavage (BAL) fluids, aprotinin (1000 KIU/ml) present in the lavaging solution. Four groups of ten guinea-pigs were used. In each group, nine were passively sensitized with anti-ovalbumin (OA) serum, and one received control serum (CON). 24 h later, they received mepyramine (0.5 mg/kg, i.p.) and 30 min later they inhaled aerosols of OA (2% in saline) (OA/OA, $n = 4$), saline (OA/SAL, $n = 4$), or LPS (OA/LPS, $n = 1$) and control serum treated animals inhaled LPS (CON/LPS, $n = 1$). 30, 60, 90 and 120 min post-challenge, animals were anaesthetized and bronchial alveolar lavage performed. Each number is the mean ± SEM

| Treatment | Time (min) post challenge | | | |
|---|---|---|---|---|
| | 30 | 60 | 90 | 120 |
| OA/OA | ND | 6 ± 3 | 18 ± 3 | 26 ± 6 |
| OA/SAL | ND | ND | ND | ND |
| OA/LPS | 8 ± 3 | 12 ± 8 | 63 ± 15 | 40 ± 10 |
| CON/LPS | 9 ± 4 | 15 ± 9 | 56 ± 6 | 36 ± 13 |

ND = None detected.

fluid collected 30 min post-challenge, but increasing amounts of TNF-α were detected at 60, 90 and 120 min post-challenge and were correlated positively with time BAL fluid was collected post-challenge. By contrast, TNF-α was not detected in BAL fluid from animals challenged with only saline. However, TNF-α was detected in BAL fluid from both positive controls, the passively sensitized and control serum treated animals that had inhaled LPS aerosol (Table 1, Fig. 1). The cytotoxicity of this TNF-α was prevented if the BAL fluid, rabbit anti-human TNF-α serum (5 μl) and the L929 cells were incubated together; rabbit pre-immune serum (5 μl) was without effect on the cytotoxicity. (BAL at 90 min: OA/OA: + preimmune serum, [TNF-α] = 21 ± 8 U/ml; + anti-TNF-α serum, [TNF-α] = <2.0 U/ml; OA/LPS: + preimmune serum, [TNF-α] = 71 ± 10 U/ml; + anti-TNF-α serum, [TNF-α] = <2.0 U/ml.)

*IL-1 and IL-6:* Neither IL-1 nor IL-6 could be detected in BAL fluid as the latter was toxic to the cell lines used to assay for the cytokines. Dialysis of the BAL fluids for 24 h failed to completely eliminate this toxicity.

*Acute effects of inhalation of OA, saline and LPS aerosols in guinea-pigs:* In all guinea-pigs that were passively sensitized to OA and inhaled OA aerosols, some signs of anaphylaxis were noted despite the presence of mepyramine. Animals were carefully observed and those that showed signs of respiratory distress were immediately removed from the exposure chamber to prevent progression to severe respiratory distress and death. Exposure times in the OA/OA groups ranged from 0.5 to 8 min. Mean exposure time was 4.4 ± 0.7 min ($n = 20$). Duration of exposure to OA aerosol did not correlate with the amount of TNF-α detected in BAL fluid.

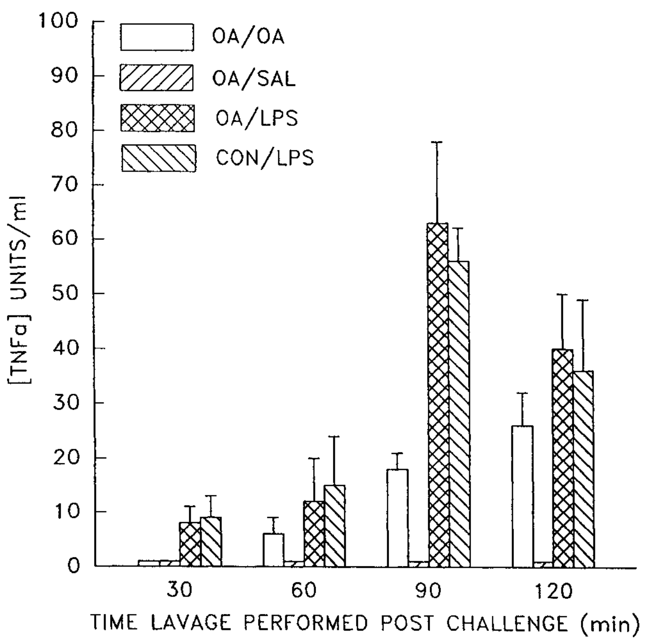

FIG. 1. Amounts of TNF-α detected in bronchial alveolar lavage (BAL) fluid at 30, 60, 90 and 120 min after challenge of guinea-pigs passively sensitized to ovalbumin (OA) with aerosols of OA (2% in saline) (OA/OA, $n = 4$) saline (OA/SAL, $n = 4$) or LPS (OA/LPS, $n = 1$) and a control serum treated animals challenged with LPS (CON/LPS, $n = 1$).

### Discussion

We report the detection of TNF-α-like activity in BAL fluid obtained from guinea-pigs passively sensitized to OA and challenged with OA aerosols. TNF-α-like activity could not be detected at 30 min post-challenge, but it was measurable (8–26 U/ml) in fluid obtained at 60, 90 and 120 min post challenge. As rabbit anti-human TNF-α antibodies prevented the cytotoxicity of the lavage fluids, it is highly likely that it is TNF-α. TNF-α levels in BAL fluids from animals that had inhaled LPS appeared to peak about 90 min post-aerosol inhalation. It is worth noting that these guinea-pigs inhaled LPS aerosol for 40 min compared to OA aerosol for up to 8 min in the other group. Thus, as we have not defined the peak of TNF-α secretion into BAL fluid for OA challenged animals, we cannot say whether 120 min represents the maximal concentration that may be achieved or whether its pattern of secretion follows that seen after LPS inhalation. The levels of TNF-α detected in these experiments are similar to those found by others in BAL fluid from guinea-pigs after inhalation of LPS[13] or cotton dust.[14]

Unlike Broide *et al.*,[9] who measured 'resting' TNF-α levels in BAL fluid from symptomatic and asymptomatic asthmatics, but like Gosset *et al.*,[8] we could not detect TNF-α unless a protease inhibitor was present in the lavaging solution. This is noteworthy. All the passively sensitized guinea-pigs showed signs of anaphylaxis upon inhalation of OA aerosols, but the duration of exposure to the OA aerosol did not appear to influence the amount of TNF-α detected in BAL fluid. Others[14,15] have shown that antigen challenge of sensitized animals leads not only to the release of a variety of mediators, but also to the release of proteases that could metabolize cytokines such as TNF-α. Thus, as BAL was performed 30–120 min after challenge, lack of aprotinin in the lavaging solution could have allowed metabolism of any TNF-α released and thus prevented its detection. This finding emphasizes the importance of adding antiproteases to lavaging solutions to help prevent breakdown of cytokines during and after BAL.

Others[6,7,17] have reported that IgE dependent reactions result in TNF-α formation and secretion. In these experiments, the sensitizing antibody is most likely to be a subtype of IgG, probably $IgG_1$ or $IgG_2$, as passive cutaneous anaphylaxis tests (data not shown) revealed little or no IgE in the serum used to passively sensitize animals. These findings show that passive sensitization with anti-OA antibodies of the IgG class followed by antigen challenge can induce directly or indirectly the synthesis and secretion of TNF-α. The production of TNF-α is not unique to a particular cell type, but a major source of TNF-α is macrophages.[2] Gosset *et al.*[8,17] showed that alveolar macrophages collected from antigen challenged asthmatics undergoing a late reaction generated significantly more TNF-α than similar patients who underwent only an early reaction or who had experienced no reaction. We speculate that, in our experiments in guinea-pigs, most of the TNF-α that is detectable is secreted from alveolar macrophages. Neither IL-1 nor IL-6 could be detected as the lavage fluid was toxic to the cell types used in the bioassay of these cytokines. Preliminary experiments (data not shown) showed that ELISAs for murine IL-1 and IL-6 could not demonstrate the presence of these cytokines in BAL fluid. Thus, whether these cytokines are present in physiologically significant amounts in BAL fluid remains to be determined.

The physiological significance of TNF-α release following passive sensitization and antigen challenge is unclear. Our data suggest that the release of proteases and other mast cell associated mediators during anaphylaxis may act to reduce TNF-α's pro-inflammatory effects. Gosset *et al.*[18] reported that human alveolar macrophages from asthmatics have an enhanced capacity to produce TNF-α, compared with cells from normal control subjects. Thus, TNF-α may have a greater role in the chronic inflammatory processes in asthma than was previously supposed.

We conclude that TNF-α is released in passively sensitized guinea-pigs' lungs soon after antigen inhalation. A protease inhibitor must be present in the lavaging solution to enable TNF-α's detection.

### References

1. Brown PH, Crompton GK, Greening AP. Proinflammatory cytokines in acute asthma. *Lancet* 1990; **338**: 590–593.
2. Camussi G, Albano E, Tetta C, *et al.* The molecular action of tumor necrosis factor-α. *Eur J Biochem* 1991; **202**: 3–14.
3. Schollmeier K. Immunologic and pathophysiologic role of tumor necrosis factor. *Am J Respir Cell Mol Biol* 1991; **3**: 11–12.
4. Semenzato G. Tumour necrosis factor: a cytokine with multiple biological activities. *Br J Cancer* 1990; **61**: 354–361.
5. Kings MA, Chapman I, Kristersson A, *et al.* Human recombinant lymphokines and cytokines induce pulmonary eosinophilia in the guinea-pig which is inhibited by ketotifen and AH 21-132. *Int Arch Allergy Appl Immunol* 1990; **91**: 354–361.
6. Ohno I, Ohkawara Y, Yamauchi K, *et al.* Production of tumor necrosis factor with IgE receptor triggering from sensitized lung tissue. *Am J Respir Cell Mol Biol* 1991; **3**: 285–289.
7. Wershil BK, Wang Z-S, Gordon JR, *et al.* Recruitment of neutrophils during IgE-dependent cutaneous late phase reactions in the mouse is mast cell-dependent. Partial inhibition of the reaction with antiserum against tumor necrosis factor-alpha. *J Clin Invest* 1991; **87**: 446–453.
8. Gosset P, Tsicopoulos A, Wallaert B, *et al.* Increased secretion of tumor necrosis factor α and interleukin-6 by alveolar macrophages consecutive to the development of the late asthmatic reaction. *J Allergy Clin Immunol* 1991; **88**: 561–571.
9. Broide DH, Lotz M, Cuomo AJ, *et al.* Cytokines in symptomatic asthma airways. *J Allergy Clin Immunol* 1992; **89**: 958–967.
10. Hogan MM, Vogel SN. Measurement of tumor necrosis factor α and β. In: Coligan E, Kruisbeek AM, Marguiles DH, *et al.* eds. *Current Protocols in Immunology.* New York: John Wiley & Sons, 1991; 6.10.1–6.10.3.
11. Nordan RP. Measurement of mouse and human interleukin 6. In: Coligan E, Kruisbeek AM, Marguiles DH, *et al.* eds. *Current Protocols in Immunology.* New York: John Wiley & Sons, 1991; 6.10.1–6.10.3.
12. Muegge K, Durum SK. Measurement of soluble and membrane-bound interleukin-1. In: Coligan E, Kruisbeek AM, Marguiles DH, *et al.* eds. *Current Protocols in Immunology.* New York: John Wiley & Sons, 1992; 6.2.1–6.2.4.
13. De Rochemontieux-Galve B, Marchat-Amoruso B, Dayer J-M, *et al.* Tumor necrosis factor and interleukin-1 activities in free lung cells after single and repeated inhalation of bacterial endotoxin. *Infect Immunity* 1991; **59**: 3646–3650.
14. Ryan LK, Karol MH. Release of tumor necrosis factor in guinea-pigs after inhalation of cotton dust. *Am J Respir Cell Mol Biol* 1991; **5**: 93–98.
15. Miller HRP, Woodbury RG, Huntley JF, *et al.* Systemic release of mucosal mast-cell protease in primed rats challenged with *Nippostrongylus brasiliensis. Immunology* 1983; **49**: 471–479.
16. Caughey GH, Lazarus SC, Viro NF, *et al.* Tryptase and chymase: comparison of extraction and release in two dog mastocytoma lines. *Immunology* 1988; **63**: 339–344.
17. Borish L, Mascali JJ, Rosenwasser LJ. IgE-dependent cytokine production by human peripheral blood mononuclear phagocytes. *J Immunol* 1991; **147**: 63–67.
18. Gosset P, Tsicopoulos A, Wallaert B, *et al.* Tumor necrosis factor alpha and interleukin-6 production by human mononuclear phagocytes from allergic asthmatics after IgE-dependent stimulation. *Am Rev Respir Dis* 1992; **146**: 768–774.

ACKNOWLEDGEMENTS. This work was supported by the Medical Research Council of Canada, the Alberta Lung Association, the Natural Sciences and Engineering Research Council of Canada, the Fond de Recherche en Santé du Québec and the Association Pulmonaire du Québec. D.K. thanks the Alberta Lung Association for a Graduate Scholarship. We thank Marie Bédard for processing the data and for the statistical analyses.

**Received 2 September 1992; accepted in revised form 5 October 1992**
